# Drimane sesquiterpenoids from a wetland soil-derived fungus *Aspergillus* *calidoustus* TJ403-EL05

**DOI:** 10.1007/s13659-022-00349-w

**Published:** 2022-07-22

**Authors:** Sitian Zhang, Shuyuan Mo, Fengli Li, Yaxin Zhang, Jianping Wang, Zhengxi Hu, Yonghui Zhang

**Affiliations:** Hubei Key Laboratory of Natural Medicinal Chemistry and Resource Evaluation, School of Pharmacy, Tongji Medical College, Huazhong University of Science and Technology, Wuhan, 430030 China

**Keywords:** *Aspergilluscalidoustus*, Drimane sesquiterpenoids, Structure elucidation, Anti-inflammatory activity

## Abstract

Soil-derived fungi represent an insufficiently tapped reservoir for discovering new and bioactive natural products (NPs), and despite an ever-increasing number of unknown NPs have been discovered over the past few decades, much of the hidden biosynthetic potential is still in an urgent need to be disclosed. In this research, a chemical investigation was performed on a wetland soil-derived fungus *Aspergillus* *calidoustus* TJ403-EL05, leading to the isolation of a total of fourteen drimane sesquiterpenoids (**1**–**14**), incorporating three new ones, namely ustusols F–H (**1**–**3**). Their structures, comprising absolute configurations, were completely authenticated by widespread spectroscopic data, quantum chemical ^13^C NMR and ECD calculations, and X-ray crystallography experiments. Compound **14** exhibited moderate anti-inflammatory activity by inhibiting the LPS-induced NO release (IC_50_ = 25.6 μM).

**Graphical Abstract:**

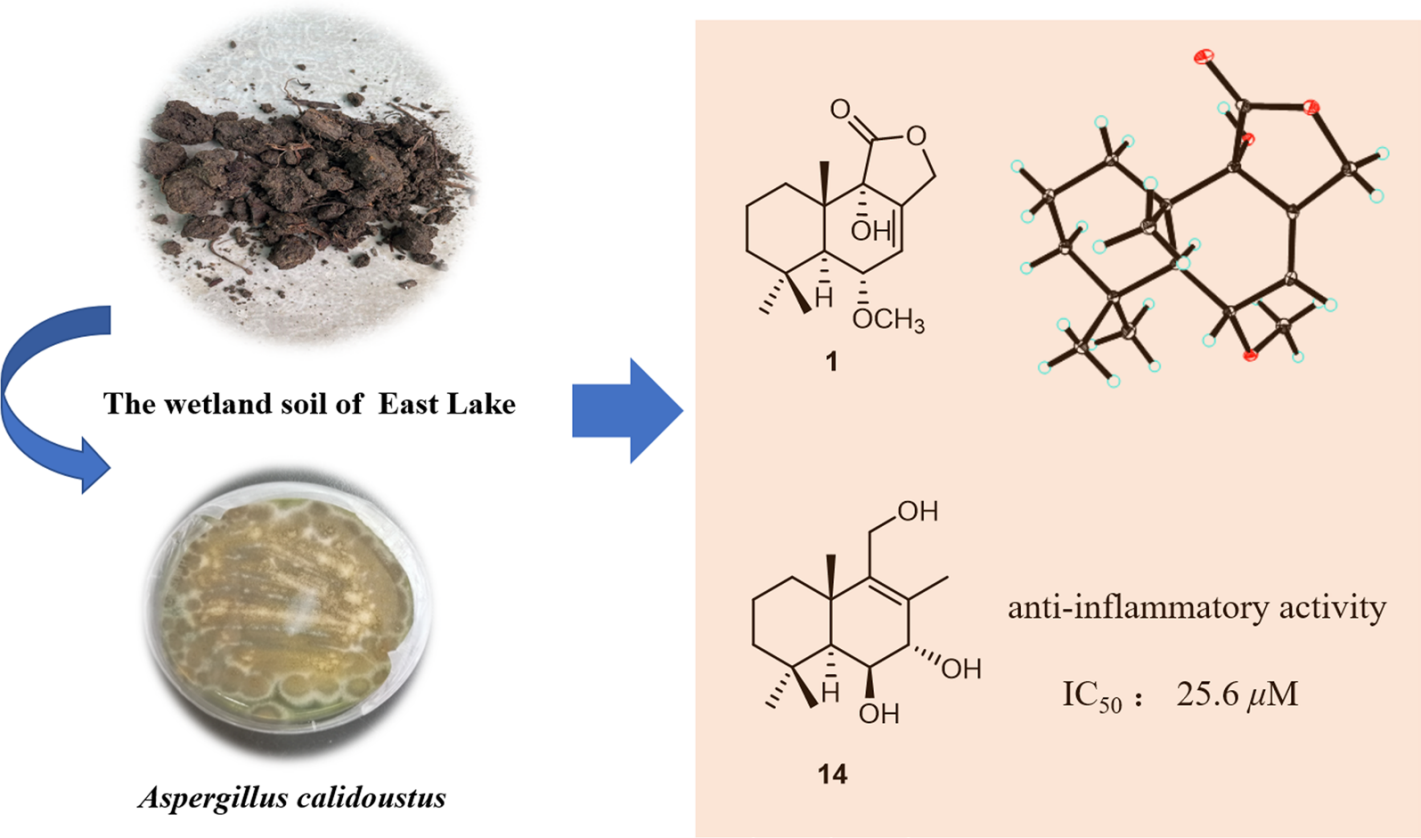

**Supplementary Information:**

The online version contains supplementary material available at 10.1007/s13659-022-00349-w.

## Introduction

Over the past few decades, a large proportion of medicines originate from various natural resources, especially from the field of microbiology [1]. Terrestrial microorganisms have a huge biosynthetic capacity to produce structurally diverse and pharmacologically active NPs, which have become important chemical entities in drug discovery [2]. For example, cyclosporine, isolated from the soil-derived *Tolypocladium* *inflatum*, was the first immunosuppressive agent to enable selective immune regulation of T cells, without excessive toxicity [3]. The discovery of cyclosporine in 1971 initiated a new era in the immunopharmacology field, and is still widely used in clinical practice. Therefore, soil-derived fungi have attracted, and will attract increasing attention in the NPs-related research fields.

**Fig. 1:**
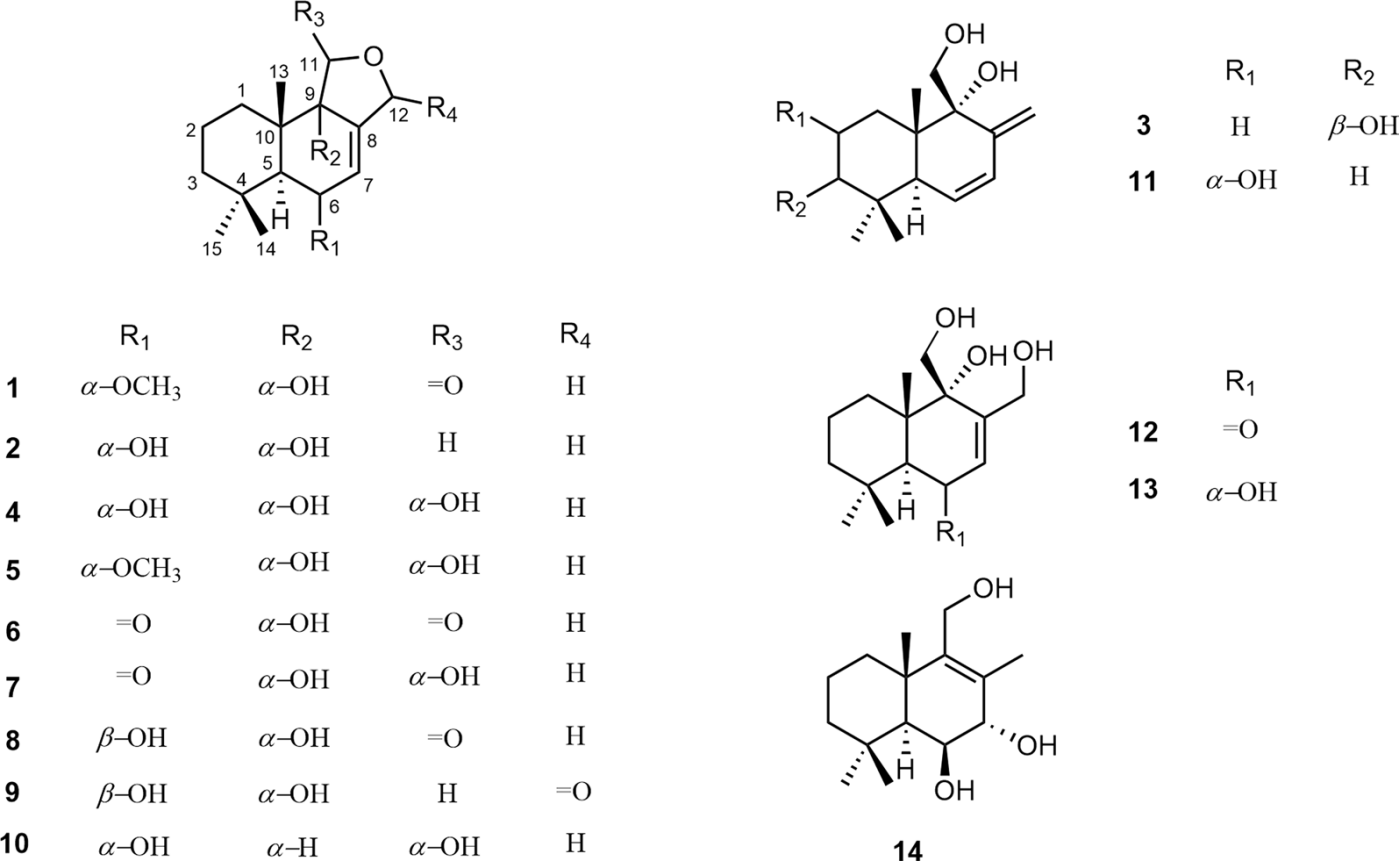
Chemical structures of compounds **1–14**

Aimed at searching for structurally unique and pharmacologically attractive NPs from the soil-derived fungi [4–6], strain *A.* *calidoustus* TJ403-EL05 that was separated from a wetland soil collected from East Lake in Wuhan City, caught our attention and was thus chemically investigated, which afforded three new drimane sesquiterpenoids, namely ustusols F–H (**1**–**3**), and eleven known congeners (**4**–**14**). In this paper, the isolation, structural characterization, and anti-inflammatory activity of these drimane sesquiterpenoids (Fig. 1) were elaborated.

## Results and discussion

**Fig. 2:**
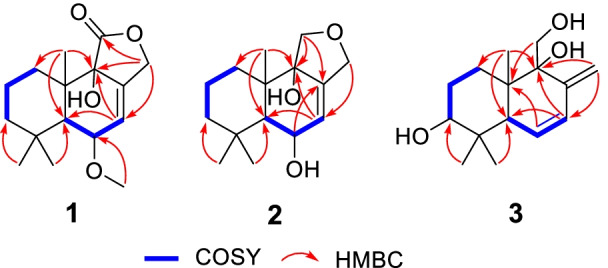
Key ^1^H–^1^H COSY and HMBC correlations of compounds **1**–**3**

**Fig. 3:**
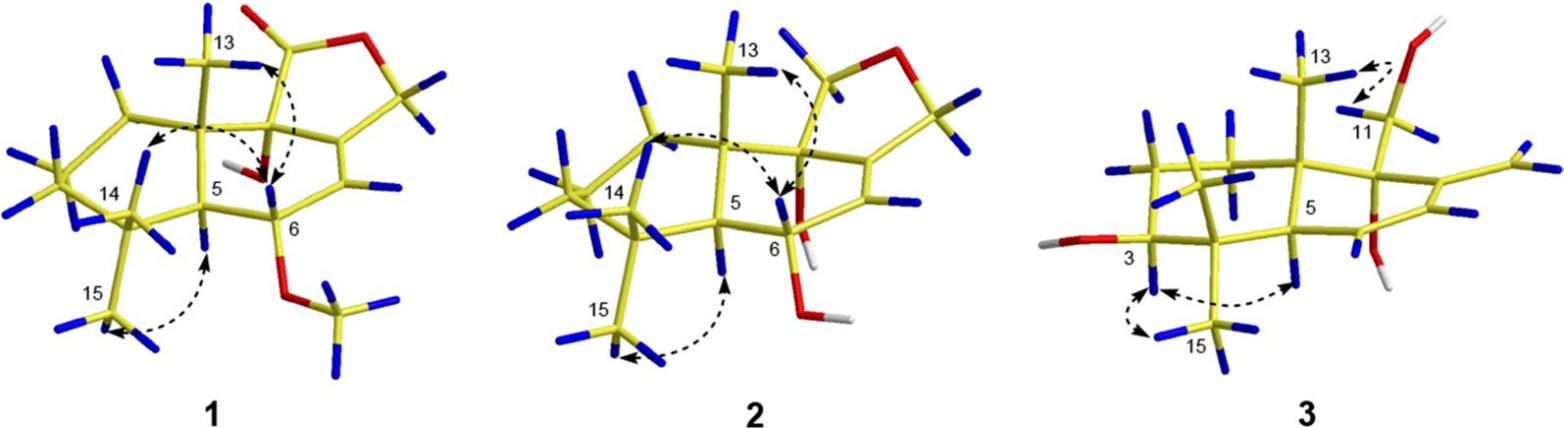
Key NOESY correlations (dashed black arrows) of compounds **1**–**3**

**Fig. 4:**
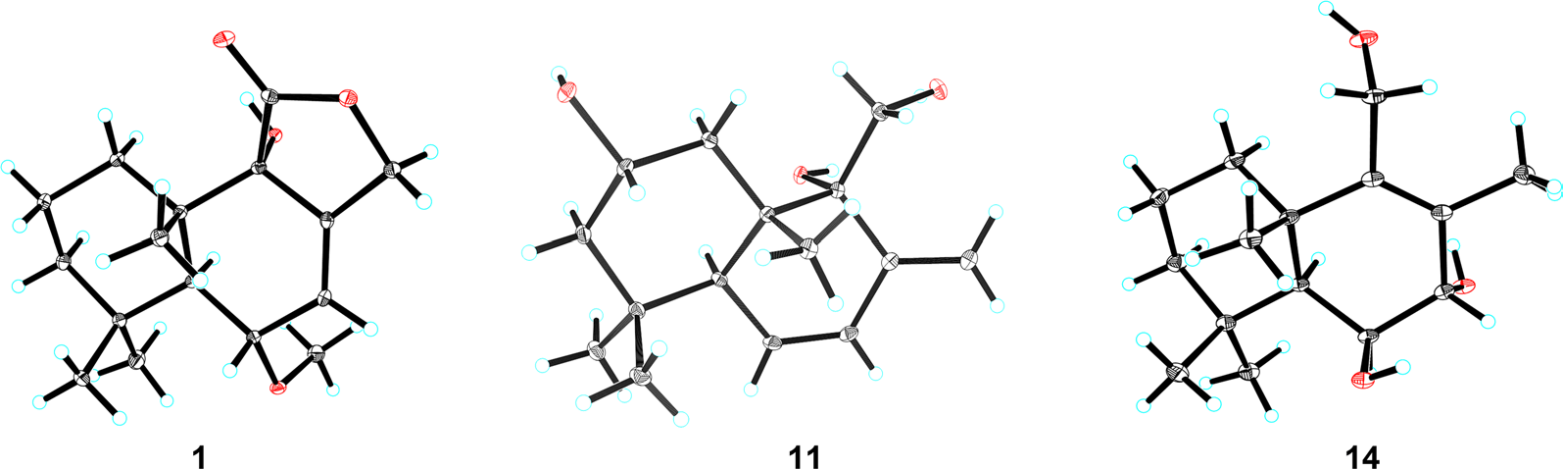
X-ray crystallographic structures of **1**, **11**, and **14**

Compound **1** was purified as a colorless crystal. According to the HRESIMS analysis showing a sodium adduct ion at *m*/*z* 303.1567 (calcd for 303.1567), its molecular formula was determined as C_16_H_24_O_4_, implying 5 degrees of unsaturation. The ^1^H NMR data (Table 1) of **1** showed obvious signals as three methyl protons (*δ*_H_ 1.08, 1.04, and 0.90), four methylene protons (*δ*_H_ 4.97/4.70, 1.62/2.08, 1.42/1.33, and 1.58), three methine protons (*δ*_H_ 6.05, 3.97, and 1.98) and one methoxy proton (*δ*_H_ 3.31). With the help of DEPT and HSQC spectroscopic analyses, the ^13^C NMR data (Table 1) of **1** demonstrated the existence of 16 carbon signals that were attributable to three methyls (*δ*_C_ 35.0, 23.1 and 18.5), one methoxy (*δ*_C_ 54.4), four methylenes (*δ*_C_ 69.0, 43.1, 30.5, and 18.4), three methines (*δ*_C_ 125.2, 76.3, and 46.1), four quaternary carbons (*δ*_C_ 135.6, 74.6, 41.7 and 33.8) and one ester carboxyl carbon (*δ*_C_ 175.2). The 1D and 2D NMR data of **1** were highly similar to those of the known 9*α*-hydroxy-5*α*-drim-7-ene-6-one-11,12-olide (**6**) [7], uncovering **1** and **6** to possess the same drimane sesquiterpenoid core skeleton. The significant difference of **1** and **6** was the existence of one methoxy group linked at C-6 in **1** instead of a conjugated ketone carbonyl (C-6) in **6**, as further supported based on the key HMBC correlations (Fig. 2) of 6-OMe (*δ*_H_ 3.31) with C-6 (*δ*_C_ 76.3) and of H-6 (*δ*_H_ 3.97) with C-5 (*δ*_C_ 46.1), C-7 (*δ*_C_ 125.2), and C-8 (*δ*_C_ 135.6). In the NOESY experiment (Fig. 3), the key NOE correlations of H-6 with Me-14 (*δ*_H_ 1.04)/Me-13 (*δ*_H_ 0.90) and of H-5 (*δ*_H_ 1.98) with Me-15 (*δ*_H_ 1.08) suggested that H-6, Me-13 and Me-14 should all be *β*-oriented, while H-5 and Me-15 were all *α*-oriented. However, no useful NOE signals could be applied to verify the configuration of C-9. Fortunately, a suitable crystal of **1** was acquired by recrystallization and then furnished for X-ray crystallographic experiment (Fig. 4). According to a Flack parameter of 0.01(3), the absolute configuration of **1** was unequivocally confirmed as 5*S*, 6*S*, 9*S*, and 10*S*. Accordingly, the absolute structure of **1**, named as ustusol F, was defined.

**Table 1:** NMR data of **1**–**3** (*δ* in ppm, *J* in Hz)

No.	**1 (In CDCl**_3_)		**2 (In DMSO-***d*_6_)		**3 (In CDCl**_3_)	
	*δ*_H_^a^	*δ*_C_^b^	*δ*_H_^a^	*δ*_C_^b^	*δ*_H_^a^	*δ*_C_^b^
1	1.62 m; 2.08 m	30.5 CH_2_	1.39 m; 1.61 m	32.3 CH_2_	1.43 m; 1.74 m	29.5 CH_2_
2	1.58 m	18.4 CH_2_	1.39 m; 1.45 m	18.3 CH_2_	1.62 m; 1.74 m	27.6 CH_2_
3	1.33 m; 1.42 m	43.1 CH_2_	1.14 m; 1.26 m	43.3 CH_2_	3.33 m	78.2 CH
4		33.8 C		32.9 C		38.6 C
5	1.98 d (9.0)	46.1 CH	1.71 d (10.2)	48.3 CH	2.44 t (3.0, 1.7)	46.8 CH
6	3.97 m	76.3 CH	4.02 m	67.1 CH	5.79 dd (10.2, 1.7)	129.1 CH
7	6.05 m	125.2 CH	5.65 d (1.7)	131.9 CH	6.11 dd (10.2, 3.0)	129.5 CH
8		135.6 C		138.2 C		145.6 C
9		74.6 C		74.6 C		76.1 C
10		41.7 C		42.0 C		41.3 C
11		175.2 C	3.45 d (14.5)	62.0 CH_2_	3.82 d (11.0); 3.93 dd (11.0, 4.7)	62.1 CH_2_
12	4.70 dt (12.4, 2.0); 4.97 dt (12.4, 2.0)	69.0 CH_2_	4.04 d (11.0)	61.5 CH_2_	5.03 d (1.4); 5.48 d (1.4)	114.8 CH_2_
13	0.90 s	18.5 CH_3_	0.90 s	17.5 CH_3_	0.75 s	16.4 CH_3_
14	1.04 s	23.1 CH_3_	1.00 s	22.9 CH_3_	0.84 s	16.7 CH_3_
15	1.08 s	35.0 CH_3_	1.10 s	36.6 CH_3_	1.10 s	28.2 CH_3_
OCH_3_	3.31 s	54.4 CH_3_				

^a^Recorded at 400 MHz

^b^Recorded at 100 MHz

“m” means overlapped or multiplet with other signals

**Fig. 5:**
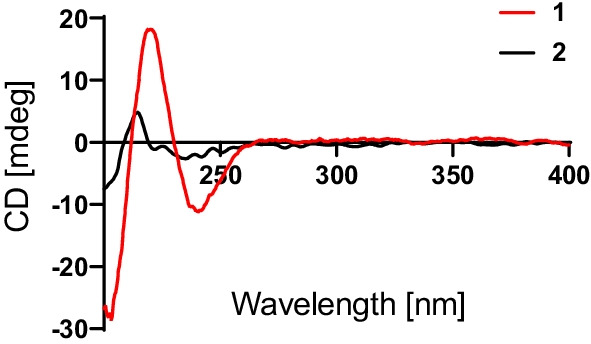
Experimental ECD curves of compounds **1** and **2** in MeOH

Compound **2**, obtained as a white powder, was determined to possess a molecular formula of C_15_H_24_O_3_, as evidenced by its positive HRESIMS data at *m/z* 275.1618 (calcd for C_15_H_24_O_3_Na^+^, 275.1618). By comparing its ^1^H, ^13^C, and DEPT NMR data (Table 1) with those of the known 6-*epi*-pereniporin A (**4**) [8], we could speculate that both compounds were structural analogues, with the only distinction being that one hydroxy group linked at C-11 was absent in **2**, as fully supported by the HMBC correlations (Fig. 2) of H_2_-11 (*δ*_H_ 3.45) with C-8 (*δ*_C_ 138.2), C-9 (*δ*_C_ 74.6), and C-12 (*δ*_C_ 61.5). Similar NOESY data (Fig. 3) and ECD curves (Fig. 5) between **1** and **2** proved that these two compounds possessed the identical absolute configuration. Accordingly, the absolute structure of **2**, named as ustusol G, was defined.

**Fig. 6:**
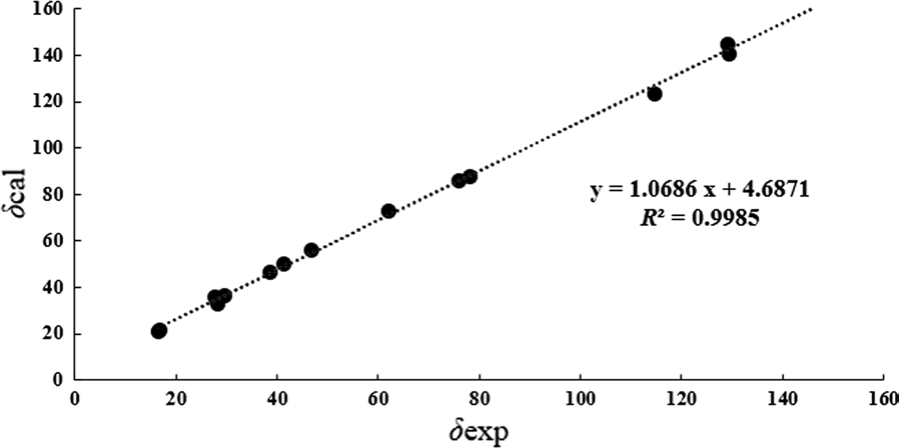
Linear correlation between the experimental and calculated ^13^C NMR chemical shifts for **3**

**Fig. 7:**
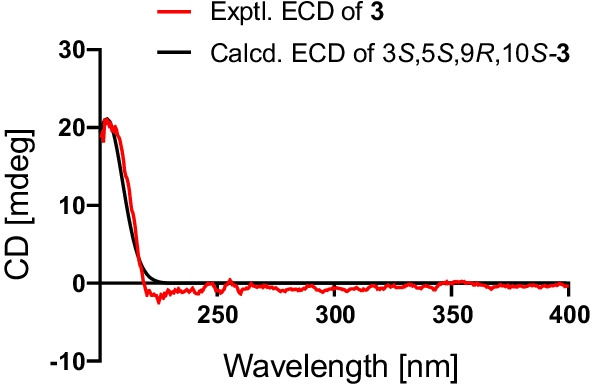
Experimental and calculated ECD spectra of compound **3**

Compound **3** was deduced to have a molecular formula of C_15_H_24_O_3_, as evidenced via its HRESIMS data. By comparing the 1D NMR data (Table 1) of **3** to those of the known ustusol D (**11**) [9] (Fig. 1) whose absolute structure was verified via crystallography experiment (Fig. 4), it revealed that both compounds possessed the identical drimane sesquiterpenoid core skeleton, with the only exception that a hydroxy group was linked at C-2 in **11** by C-3 in **3**. This conclusion was further corroborated via the key ^1^H–^1^H COSY cross-peaks of H_2_-1/H_2_-2/H-3, as well as the HMBC correlations of both Me-14 and Me-15 with C-3, C-4, and C-5 (Fig. 2). The NOE cross-peaks (Fig. 3) of Me-15*α*/H-3/H-5 and H_2_-11*/*Me-13*β* demonstrated that OH-3 was *β*-oriented in **3**. To validate this speculation, the ^13^C NMR chemical shifts of **3** were predicted at the B972/pcSseg-2 level showing the correlation coefficient (*R*^2^) value of 0.9985 (Fig. 6), which completely supported our proposed relative structure. Lastly, the quantum chemical electronic circular dichroism (ECD) calculation was employed for **3**. To our expectation, the calculated ECD plot was closely similar to the experimental one (Fig. 7), proclaiming its absolute configuration as 3*S*, 5*S*, 9*R*, and 10*S*, and this compound was named as ustusol H.

Apart from new compounds **1**–**3**, eleven known congeners were also isolated from *A.* *calidoustus* TJ403-EL05 and identified as 6-*epi*-pereniporin A (**4**) [8], 6-*epi*-*O*-methyl-pereniporin A (**5**) [8], 9*a-*hydroxy-5*a*-drim-7-ene-6-one-11,12-olide (**6**) [7], 6-dehydroxy-6-oxopereniporin A (**7**) [8], strobilactone A (**8**) [10], pereniporin B (**9**) [11], dendocarbin C (**10**) [12], ustusol D (**11**) [9], 9*a*,11,12-trihydroxydrim-7-en-6-one (**12**) [13], 12-hydroxyalbrassitriol (**13**) [14] and drim-8-en-6*β*,7*a*,11-triol (**14**) [15], by comparison of their HRESIMS and NMR data with those reported in the literature.

In the bioactivity assay, due to the limited amounts of **3**, other compounds (**1–2** and **4–14**) were tested for anti-inflammatory activity by using LPS-induced murine macrophages RAW264.7 cells. As a result, only compound **14** was found to show an inhibitory effect against the NO release (IC_50_ = 25.6 μM), and the remaining compounds did not exhibit significant activity with IC_50_ values of > 40 µM (positive control MG132: IC_50_ = 0.32 µM).

## Supplementary Information

**Additional file 1.** Supplementary figures and tables.
