# Supplementary material for: Drimane sesquiterpenoids from a wetland soil-derived fungus Aspergillus calidoustus TJ403-EL05
Source: Nat Prod Bioprospect. 2022 Jul 22;12(1):27. doi: 10.1007/s13659-022-00349-w (PMC9304466; doi:10.1007/s13659-022-00349-w)
Supplement: Supplementary file 1 — Additional file 1. Supplementary figures and tables. [file 13659_2022_349_MOESM1_ESM.doc]

Supporting Information

**Drimane sesquiterpenoids from a wetland soil-derived fungus *Aspergillus calidoustus* TJ403-EL05**

Sitian Zhang^†^, Shuyuan Mo^†^, Fengli Li, Yaxin Zhang, Jianping Wang, Zhengxi Hu^*^ and Yonghui Zhang^*^

*Hubei Key Laboratory of Natural Medicinal Chemistry and Resource Evaluation, School of Pharmacy, Tongji Medical College, Huazhong University of Science and Technology, Wuhan 430030, China*

**Contents of Supporting Information**

[**Figure S1.** ^1^H NMR spectrum of compound **1** (Recorded in CDCl_3_) 3](#__RefHeading___Toc104810964)

[**Figure S2.** ^13^C NMR and DEPT spectrum of compound **1** (Recorded in CDCl_3_) 4](#__RefHeading___Toc104810965)

[**Figure S3.** HSQC spectrum of compound **1** (Recorded in CDCl_3_) 5](#__RefHeading___Toc104810966)

[**Figure S4.** HMBC spectrum of compound **1** (Recorded in CDCl_3_) 6](#__RefHeading___Toc104810967)

[**Figure S5.** ^1^H–^1^H COSY spectrum of compound **1** (Recorded in CDCl_3_) 7](#__RefHeading___Toc104810968)

[**Figure S6.** NOESY spectrum of compound **1** (Recorded in CDCl_3_) 8](#__RefHeading___Toc104810969)

[**Figure S7.** HRESIMS spectrum of compound **1** 9](#__RefHeading___Toc104810970)

[**Figure S8.** UV spectrum of compound **1** 10](#__RefHeading___Toc104810971)

[**Figure S9.** IR spectrum of compound **1** 11](#__RefHeading___Toc104810972)

[**Figure S10.** ^1^H NMR spectrum of compound **2** (Recorded in DMSO-*d*_6_) 12](#__RefHeading___Toc104810973)

[**Figure S11.** ^13^C NMR and DEPT spectrum of compound **2** (Recorded in DMSO-*d*_6_) 13](#__RefHeading___Toc104810974)

[**Figure S12.** HSQC spectrum of compound **2** (Recorded in DMSO-*d*_6_) 14](#__RefHeading___Toc104810975)

[**Figure S13.** HMBC spectrum of compound **2** (Recorded in DMSO-*d*_6_) 15](#__RefHeading___Toc104810976)

[**Figure S14.** ^1^H–^1^H COSY spectrum of compound **2** (Recorded in DMSO-*d*_6_) 16](#__RefHeading___Toc104810977)

[**Figure S15.** NOESY spectrum of compound **2** (Recorded in DMSO-*d*_6_) 17](#__RefHeading___Toc104810978)

[**Figure S16.** HRESIMS spectrum of compound **2** 18](#__RefHeading___Toc104810979)

[**Figure S17.** UV spectrum of compound **2** 19](#__RefHeading___Toc104810980)

[**Figure S18.** IR spectrum of compound **2** 20](#__RefHeading___Toc104810981)

[**Figure S19.** ^1^H NMR spectrum of compound **3** (Recorded in CDCl_3_) 21](#__RefHeading___Toc104810982)

[**Figure S20.** ^13^C NMR and DEPT spectrum of compound **3** (Recorded in CDCl_3_) 22](#__RefHeading___Toc104810983)

[**Figure S21.** HSQC spectrum of compound **3** (Recorded in CDCl_3_) 23](#__RefHeading___Toc104810984)

[**Figure S22.** HMBC spectrum of compound **3** (Recorded in CDCl_3_) 24](#__RefHeading___Toc104810985)

[**Figure S23.** ^1^H–^1^H COSY spectrum of compound **3** (Recorded in CDCl_3_) 25](#__RefHeading___Toc104810986)

[**Figure S24.** NOESY spectrum of compound **3** (Recorded in CDCl_3_) 26](#__RefHeading___Toc104810987)

[**Figure S25.** HRESIMS spectrum of compound **3** 27](#__RefHeading___Toc104810988)

[**Figure S26.** UV spectrum of compound **3** 28](#__RefHeading___Toc104810989)

[**Figure S27.** IR spectrum of compound **3** 29](#__RefHeading___Toc104810990)

[**Experimental** 30](#__RefHeading___Toc104810991)

[**^13^C NMR Calculation Data of 3** 35](#__RefHeading___Toc104810992)

[**ECD Calculation Data of 3** 48](#__RefHeading___Toc104810993)

**Figure S1.** ^1^H NMR spectrum of compound **1** (Recorded in CDCl_3_)

**Figure S2.** ^13^C NMR and DEPT spectrum of compound **1** (Recorded in CDCl_3_)

**Figure S3.** HSQC spectrum of compound **1** (Recorded in CDCl_3_)

**Figure S4.** HMBC spectrum of compound **1** (Recorded in CDCl_3_)

**Figure S5.** ^1^H–^1^H COSY spectrum of compound **1** (Recorded in CDCl_3_)

**Figure S6.** NOESY spectrum of compound **1** (Recorded in CDCl_3_)

**Figure S7.** HRESIMS spectrum of compound **1**

**Figure S8.** UV spectrum of compound **1**

**Figure S9.** IR spectrum of compound **1**

**Figure S10.** ^1^H NMR spectrum of compound **2** (Recorded in DMSO-*d*_6_)

**Figure S11.** ^13^C NMR and DEPT spectrum of compound **2** (Recorded in DMSO-*d*_6_)

**Figure S12.** HSQC spectrum of compound **2** (Recorded in DMSO-*d*_6_)

**Figure S13.** HMBC spectrum of compound **2** (Recorded in DMSO-*d*_6_)

**Figure S14.** ^1^H–^1^H COSY spectrum of compound **2** (Recorded in DMSO-*d*_6_)

**Figure S15.** NOESY spectrum of compound **2** (Recorded in DMSO-*d*_6_)

**Figure S16.** HRESIMS spectrum of compound **2**

**Figure S17.** UV spectrum of compound **2**


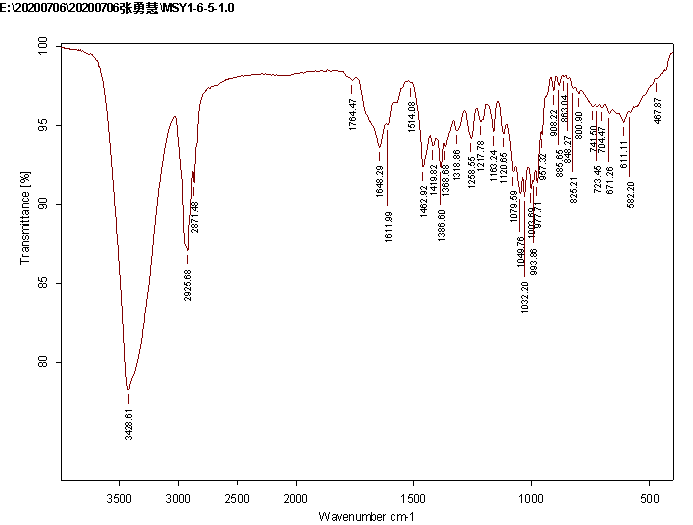


**Figure S18.** IR spectrum of compound **2**

**Figure S19.** ^1^H NMR spectrum of compound **3** (Recorded in CDCl_3_)

**Figure S20.** ^13^C NMR and DEPT spectrum of compound **3** (Recorded in CDCl_3_)

**Figure S21.** HSQC spectrum of compound **3** (Recorded in CDCl_3_)

**Figure S22.** HMBC spectrum of compound **3** (Recorded in CDCl_3_)

**Figure S23.** ^1^H–^1^H COSY spectrum of compound **3** (Recorded in CDCl_3_)

**Figure S24.** NOESY spectrum of compound **3** (Recorded in CDCl_3_)

**Figure S25.** HRESIMS spectrum of compound **3**

**Figure S26.** UV spectrum of compound **3**

**Figure S27.** IR spectrum of compound **3**

**Experimental**

*1 General*

Optical rotations, UV absorption data, experimental CD data, and IR spectra were measured with a PerkinElmer PE-341 polarimeter (PerkinElmer, Waltham, MA, USA), a PerkinElmer Lambda 35 instrument (PerkinElmer, USA) in MeOH, a JASCO-810 spectrometer (Jasco, Japan), and a Bruker Vertex 70 FT-IR instrument (Bruker, Karlsruhe, Germany), respectively. 1D and 2D NMR data were acquired on a Bruker AM-400 NMR instrument (Bruker, Germany). Chemical shifts (in ppm) are referenced to the solvent peaks (*δ*_H_ 7.26 and *δ*_C_ 77.2) for CDCl_3_ and (*δ*_H_ 2.50 and *δ*_C_ 39.5) for DMSO-*d*_6_. HRESIMS data were acquired from a Thermo Fisher LC-LTQ-Orbitrap XL instrument (Bruker Daltonics, Germany). Silica gel (200–300 mesh, Qingdao Marine Chemical, Inc., Qingdao, China), ODS (50 *μ*m, YMC Co. Ltd., Japan), and Sephadex LH-20 (GE Healthcare Bio-Sciences AB, Sweden) were applied for the column chromatography (CC). The samples were analyzed by an Agilent 1100 liquid chromatograph using an RP-C_18_ column (5 *μ*m, 10 × 250 mm, Welch Ultimate XB-C_18_). The thin-layer chromatography (TLC) analysis was carried out on precoated plates and detected with 10% H_2_SO_4_ in EtOH followed by heating.

*2*. *Fungal material*

Strain *A. calidoustus* TJ403-EL05 was separated from a wetland soil collected from the East Lake, Wuhan City, Hubei Province, China, in July 2018. The strain was identified via the morphology analysis and Internal Transcribed Spacer (ITS) sequencing data (GenBank accession number OM283555) of the rDNA. The fungal sample (HUST20180818) was stored at the culture collection center of School of Pharmacy, Tongji Medical College, Huazhong University of Science and Technology (HUST).

*3*. *Extraction and isolation*

The fungus *A. calidoustus* TJ403-EL05 was cultivated on thirty potato dextrose agar (PDA) plates to prepare the seed cultures, which were cut into a plenty of small pieces and then cultivated into 240 × 1 L Erlenmeyer flasks (ingredients: 250 g of rice and 200 mL of distilled water), by autoclaving at 121 °C for 30 min and then cooling to room temperature. After incubation for 30 days at 26 °C, the rice culture was gathered together and extracted eight times with EtOAc at room temperature to afford a crude extract (453 g). The EtOAc extract (453 g) was subjected to silica gel open glass CC with eluted with petroleum ether (PE)–EtOAc (30:1, 20:1, 10:1, 5:1, 2:1, 1:1, and 0:1, v/v) to give six major fractions (A–F).

Fraction D (36 g) was isolated by RP-C_18_ CC eluted with MeOH–H_2_O (20%, 40%, 60%, 80%, and 100%) to give seven major fractions (D1–D7). Fraction D4 (3.2 g) was subjected to silica gel open glass CC (PE–EtOAc, 10:1–1:1, v/v) and repeated RP-C_18_ HPLC (MeCN–H_2_O, 28:72, v/v; 2.0 mL/min) to give compounds **3** (1.2 mg, *t*_R_ = 24 min), **10** (20.4 mg, *t*_R_ = 22 min), **11** (13.4 mg, *t*_R_ = 28 min), and **14** (5.6 mg, *t*_R_ = 36 min).

Fraction E (25 g) was further separated on RP-C_18_ CC by stepwise gradient elution with MeOH–H_2_O (20%–100%) to afford six main fractions (E1–E6). Fraction E3 (1.5 g) was applied to silica gel open glass CC eluted with PE–EtOAc (stepwise 15:1–1:1, v/v) and further purified via RP-C_18_ HPLC (MeCN–H_2_O, 60:40, v/v; 2.0 mL/min) to give compounds **1** (8.4 mg, *t*_R_ = 35 min), **5** (8.5 mg, *t*_R_ = 15 min), **6** (14.0 mg, *t*_R_ = 32 min), and **9** (14.2 mg, *t*_R_ =38 min). Fraction E4 (750 mg) was purified via Sephadex LH-20 (CH_2_Cl_2_–MeOH, 1:1, v/v), silica gel open glass CC (PE–EtOAc, 10:1–1:1, v/v), and RP-C_18_ HPLC (MeCN–H_2_O, 42:58, v/v; 2.0 mL/min) to give compounds **2** (7.0 mg, *t*_R_ = 18 min) and **4** (20.8 mg, *t*_R_ = 20 min).

Fraction F (3.3 g) was chromatographed over RP-C_18_ CC (MeOH–H_2_O, 20%–100%) to give five main fractions (F1–F5). Fraction F3 (423.1 mg) was separated by Sephadex LH-20 CC eluted with CH_2_Cl_2_–MeOH (1:1, v/v), and further purified by reversed-phase HPLC (MeCN–H_2_O, 40:60, v/v; 2.0 mL/min) to give compounds **7** (3.7 mg, *t*_R_ = 23 min), **8** (6.9 mg, *t*_R_ = 28 min), **12** (15.1 mg, *t*_R_ = 2 min), and **13** (17.8 mg, *t*_R_ = 32 min).

Ustusol F (**1**): colorless block crystals; [*α*]25 D: –16 (*c* 0.10, MeOH); UV (MeOH) *λ*_max_ (log *ε*) = 202 (4.49) nm; ECD = Δ*ε*_202_ –4.79, Δ*ε*_220_ +3.10, Δ*ε*_240_ –1.90; IR *ν*_max_ = 3419, 3342, 2946, 2852, 1773, 1631, 1464, 1385, 1362, 1057, 1011, 638 cm^–1^; HRESIMS *m/z* 303.1567 [M + Na]^+^ (calcd for C_16_H_24_O_4_Na^+^, 303.1567); For ^1^H and ^13^C NMR data, see **Table 1**.

Ustusol G (**2**): white powder; [*α*]25 D: –18 (*c* 0.10, MeOH); UV (MeOH) *λ*_max_ (log *ε*) = 204 (4.46) nm; ECD = Δ*ε*_215_ +0.93, Δ*ε*_226_ –0.07, Δ*ε*_235_ –0.52; IR *ν*_max_ = 3429, 2926, 2871, 1648, 1463, 1387, 1259, 1163, 1050, 1032, 1067, 994, 611 cm^–1^; HRESIMS *m/z* 275.1618 [M + Na]^+^ (calcd for C_15_H_24_O_3_Na^+^, 275.1618); For ^1^H and ^13^C NMR data, see **Table 1**.

Ustusol H (**3**): white powder; [*α*]25 D: +34 (*c* 0.10, MeOH); UV (MeOH) *λ*_max_ (log *ε*) = 202 (4.39) nm; ECD = Δ*ε*_203_ +3.21, Δ*ε*_222_ –0.24; IR *ν*_max_ = 3387, 2924, 2854, 1685, 1634, 1458, 1385, 1114, 1019, 989, 743 cm^–1^; HRESIMS *m*/*z* 275.1643 [M + Na]^+^ (calcd for C_15_H_24_O_3_Na^+^, 275.1618); For ^1^H and ^13^C NMR data, see **Table 1**.

*4*. *X-ray crystal structure analysis*

Suitable crystals of compounds **1**, **11**, and **14** were obtained from MeOH–H_2_O (20:1, v/v) at 4 °C. The intensity data were collected at 100 K on a XtaLAB PRO MM007HF diffractometer using Cu K*α* radiation. Using Olex2 [1], the structures were solved by direct methods with SHELXL-2014/7 [2]. Refinements were performed with SHELXL-2014/7 refinement package via means of full-matrix least-squares on *F*^2^, with anisotropic displacement parameters used for all the non-hydrogen atoms. The hydrogen atoms were located at the calculated positions and refined with a riding model. The crystallographic data for these structures have been deposited in the Cambridge Crystallographic Data Centre (CCDC 2132161 for **1**, CCDC 2143513 for **11**, CCDC 2131805 for **14**). Copies of the data can be obtained free of charge on application to CCDC, 12 Union Road, Cambridge CB 1EZ, UK [fax: Int. + 44 (0) (1223) 336 033; e-mail: [deposi@ccdc.cam.ac.uk](mailto:deposi@ccdc.cam.ac.uk)].

*Crystallographic data for compound* ***1***: C_16_H_24_O_4_, *M* = 280.35, *a* = 7.08190(10) Å, *b* = 13.4303(2) Å, *c* = 14.9888(3) Å, *α* = 90°, *β* = 90°, *γ* = 90°, *V* = 1425.62(4) Å^3^, *T* = 100(2) K, space group *P*2_1_2_1_2_1_, *Z* = 4, *μ*(Cu K*α*) = 0.749 mm^–1^, 12763 reflections measured, 2795 independent reflections (*R_int_* = 0.0332). The final *R_1_* values were 0.0274 (*I* > 2*σ*(*I*)). The final *wR*(*F*^2^) values were 0.0708 (*I* > 2*σ*(*I*)). The final *R_1_* values were 0.0275 (all data). The final *wR*(*F*^2^) values were 0.0708 (all data). The goodness of fit on *F*^2^ was 1.105. Flack parameter = 0.01(3).

*Crystallographic data for compound* ***11***: C_15_H_24_O_3_, *M* = 252.34, *a* = 7.85579(4) Å, *b* = 8.02660(4) Å, *c* = 20.91128(11) Å, *α* = 90°, *β* = 90°, *γ* = 90°, *V* = 1318.566(12) Å^3^, *T* = 99.99(10) K, space group *P*2_1_2_1_2_1_, *Z* = 4, *μ*(Cu K*α*) = 0.691 mm^–1^, 13037 reflections measured, 2623 independent reflections (*R_int_* = 0.0225). The final *R_1_* values were 0.0259 (*I* > 2*σ*(*I*)). The final *wR*(*F*^2^) values were 0.0793 (*I* > 2*σ*(*I*)). The final *R_1_* values were 0.0261 (all data). The final *wR*(*F*^2^) values were 0.0695 (all data). The goodness of fit on *F*^2^ was 1.105. Flack parameter = –0.02(5).

*Crystallographic data for compound* ***14***: 4(C_15_H_26_O_3_)•H_2_O, *M* = 1035.44, *a* = 14.8572(8) Å, *b* = 19.8554(10) Å, *c* = 19.9184(10) Å, *α* = 90°, *β* = 90°, *γ* = 90°, *V* = 5875.8(5) Å^3^, *T* = 103(2) K, space group *P*2_1_2_1_2_1_, *Z* = 4, *μ*(Cu Kα) = 0.641 mm^–1^, 135358 reflections measured, 11172 independent reflections (*R_int_* = 0.0769). The final *R_1_* values were 0.0309 (*I* > 2*σ*(*I*)). The final *wR*(*F*^2^) values were 0.0792 (*I* > 2*σ*(*I*)). The final *R_1_* values were 0.0322 (all data). The final *wR*(*F*^2^) values were 0.0808 (all data). The goodness of fit on *F*^2^ was 1.032. Flack parameter = 0.05(3).

*5*. *Measurement of NO production in the RAW264.7 mouse macrophages induced by LPS*

The procedures and methods for the anti-inflammatory activity assay was referred to the previously reported reference [3].

References:

[1] Dolomanov OV, Bourhis LJ, Gildea RJ, Howard JAK, Puschmann H. OLEX2: a complete structure solution, refinement and analysis program. J Appl Crystallogr. 2009;42:339-341.

[2] Sheldrick GM. A short history of SHELX. Acta Crystallogr A. 2008;64:112-122.

[3] Hu Z, Sun W, Li F, Guan J, Lu Y, Liu J, Tang Y, Du G, Xue Y, Luo Z, Wang J, Zhu H, Zhang Y. Fusicoccane-derived diterpenoids from *Alternaria brassicicola*: investigation of the structure-stability relationship and discovery of an IKK*β* inhibitor. Org Lett. 2018;20:5198-5202.

**^13^C NMR Calculation Data of 3**

The plausible conformers of compound **3** were performed by using a random search in the Sybyl-X 2.0 using the MMFF94S force field with an energy cutoff of 2.5 kcal/mol [1]. Subsequently, geometry optimizations and frequency analyses were implemented using DFT at the B3LYP-D3(BJ)/6-31G* level in the vacuum using Gaussian 09 [2]. All conformers used for property calculations in this work were characterized to be stable point on potential energy surface (PES) with no imaginary frequencies. NMR shielding constants were computed using the GIAO method at the B972/pcSseg-2 level in PCM chloroform using Gaussian 09. Gibbs free energies for conformers were determined by using thermal correction at B3LYP-D3(BJ)/6-31G* level using Gaussian 09 and electronic energies evaluated at the wB97M-V/def2-TZVP level in PCM chloroform using ORCA5.0.0 [3]. To get the final chemical shifts, the chemical shifts of the conformers were averaged according to the Boltzmann distribution theory and their relative Gibbs free energy (∆G).

References:

[1] St. Louis, MO. Sybyl Software, version X 2.0; Tripos Associates Inc. 2013.

[2] Frisch MJ, Trucks GW, Schlegel HB, et al. Gaussian 09, Revision D.01, Gaussian, Inc., Wallingford CT, 2009.

[3] Neese F. The ORCA program system. WIREs Comput Mol Sci. 2012;2:73-78.

**Table S1.** Gibbs free energies^a^ and equilibrium populations^b^ of low-energy conformers of **3**.

| Conformers | ∆G (a.u.) | P(%)/100 | G (a.u.) |
| --- | --- | --- | --- |
| 1 | 0.00139 | 9.82 | -811.352083 |
| 2 | 0.00308 | 1.65 | -811.350397 |
| 3 | 0.00362 | 0.93 | -811.349854 |
| 4 | 0.0 | 42.85 | -811.353474 |
| 5 | 0.00301 | 1.77 | -811.350463 |
| 6 | 0.0 | 42.98 | -811.353476 |

^a^PBE0/def2-TZVP, in a.u.
^b^From ∆G values at 298.15 K.

**Table S2.** Cartesian coordinates for the low-energy reoptimized random research conformers of **3** at B3LYP-D3(BJ)/6-31G* level of theory.

| Conformer 1 | | Standard Orientation (a.u.) | | | |
| --- | --- | --- | --- | --- | --- |
| Center number | Atomic number | Atomic Type | X | Y | Z |
| 0 | 6 | 0 | -11.148716 | -0.479016 | -0.463534 |
| 1 | 6 | 0 | -11.700641 | 1.835924 | -2.08253 |
| 2 | 6 | 0 | -14.033756 | 3.341206 | -1.198209 |
| 3 | 6 | 0 | -16.256449 | 1.418285 | -1.021487 |
| 4 | 6 | 0 | -15.811012 | -1.010168 | 0.598885 |
| 5 | 6 | 0 | -13.390086 | -2.30454 | -0.359276 |
| 6 | 6 | 0 | -18.756366 | 2.603753 | -0.418432 |
| 7 | 6 | 0 | -20.697011 | 1.308762 | 0.565327 |
| 8 | 6 | 0 | -20.481287 | -1.343065 | 1.272819 |
| 9 | 6 | 0 | -18.196217 | -2.708397 | 0.139612 |
| 10 | 6 | 0 | -13.509585 | 4.797099 | 1.258324 |
| 11 | 6 | 0 | -14.647363 | 5.291395 | -3.27228 |
| 12 | 8 | 0 | -9.588735 | 3.531331 | -2.112386 |
| 13 | 6 | 0 | -22.154914 | -2.38637 | 2.870744 |
| 14 | 6 | 0 | -17.921262 | -5.435191 | 1.112684 |
| 15 | 8 | 0 | -19.837037 | -7.036795 | 0.0478 |
| 16 | 8 | 0 | -18.549623 | -2.878257 | -2.545242 |
| 17 | 1 | 0 | -16.443494 | 0.697726 | -2.967764 |
| 18 | 6 | 0 | -15.59754 | -0.494558 | 3.453244 |
| 19 | 1 | 0 | -9.50229 | -1.472391 | -1.251 |
| 20 | 1 | 0 | -10.596477 | 0.139172 | 1.435671 |
| 21 | 1 | 0 | -12.107948 | 1.186069 | -4.029308 |
| 22 | 1 | 0 | -13.749057 | -3.073533 | -2.252413 |
| 23 | 1 | 0 | -12.885003 | -3.895034 | 0.870959 |
| 24 | 1 | 0 | -19.018511 | 4.596215 | -0.840212 |
| 25 | 1 | 0 | -22.46938 | 2.264084 | 0.985544 |
| 26 | 1 | 0 | -15.287277 | 5.470556 | 2.079977 |
| 27 | 1 | 0 | -12.541151 | 3.668646 | 2.686179 |
| 28 | 1 | 0 | -12.31462 | 6.435854 | 0.865154 |
| 29 | 1 | 0 | -12.948529 | 6.379813 | -3.718148 |
| 30 | 1 | 0 | -15.305469 | 4.364371 | -5.004695 |
| 31 | 1 | 0 | -16.100932 | 6.63103 | -2.666027 |
| 32 | 1 | 0 | -8.136293 | 2.60729 | -2.703538 |
| 33 | 1 | 0 | -21.970645 | -4.279222 | 3.642047 |
| 34 | 1 | 0 | -23.748446 | -1.289612 | 3.561099 |
| 35 | 1 | 0 | -17.940536 | -5.529627 | 3.183539 |
| 36 | 1 | 0 | -16.141667 | -6.232183 | 0.437827 |
| 37 | 1 | 0 | -21.45959 | -6.463491 | 0.65521 |
| 38 | 1 | 0 | -19.538532 | -4.395926 | -2.816835 |
| 39 | 1 | 0 | -16.989143 | 0.88895 | 4.094938 |
| 40 | 1 | 0 | -15.895819 | -2.236468 | 4.528706 |
| 41 | 1 | 0 | -13.726451 | 0.205212 | 3.953897 |
| Conformer 2 | | Standard Orientation (a.u.) | | | |
| Center number | Atomic number | Atomic Type | X | Y | Z |
| 0 | 6 | 0 | -11.359607 | -1.006213 | -0.163704 |
| 1 | 6 | 0 | -11.53116 | 1.442074 | -1.669438 |
| 2 | 6 | 0 | -13.827272 | 3.10135 | -0.934885 |
| 3 | 6 | 0 | -16.214966 | 1.390982 | -1.09902 |
| 4 | 6 | 0 | -16.144856 | -1.159449 | 0.380678 |
| 5 | 6 | 0 | -13.753496 | -2.612632 | -0.400155 |
| 6 | 6 | 0 | -18.647395 | 2.755584 | -0.61594 |
| 7 | 6 | 0 | -20.776332 | 1.558392 | 0.056168 |
| 8 | 6 | 0 | -20.905575 | -1.169202 | 0.417121 |
| 9 | 6 | 0 | -18.585739 | -2.633816 | -0.443214 |
| 10 | 6 | 0 | -13.436439 | 4.380414 | 1.644198 |
| 11 | 6 | 0 | -14.051933 | 5.2106 | -2.928153 |
| 12 | 8 | 0 | -9.201027 | 2.813897 | -1.556057 |
| 13 | 6 | 0 | -22.992495 | -2.280358 | 1.333906 |
| 14 | 6 | 0 | -18.630382 | -5.463211 | 0.21974 |
| 15 | 8 | 0 | -18.654236 | -5.961572 | 2.873786 |
| 16 | 8 | 0 | -18.601096 | -2.684326 | -3.188034 |
| 17 | 1 | 0 | -16.276658 | 0.817914 | -3.101239 |
| 18 | 6 | 0 | -16.193208 | -0.796558 | 3.269395 |
| 19 | 1 | 0 | -9.723043 | -2.076341 | -0.850114 |
| 20 | 1 | 0 | -10.973746 | -0.566835 | 1.829299 |
| 21 | 1 | 0 | -11.747824 | 0.954788 | -3.680265 |
| 22 | 1 | 0 | -13.947765 | -3.237165 | -2.369926 |
| 23 | 1 | 0 | -13.552869 | -4.310148 | 0.77365 |
| 24 | 1 | 0 | -18.697303 | 4.795296 | -0.854022 |
| 25 | 1 | 0 | -22.499782 | 2.628851 | 0.395707 |
| 26 | 1 | 0 | -15.218878 | 5.176187 | 2.33276 |
| 27 | 1 | 0 | -12.736673 | 3.102817 | 3.107294 |
| 28 | 1 | 0 | -12.089961 | 5.942628 | 1.462317 |
| 29 | 1 | 0 | -12.226497 | 6.158887 | -3.135962 |
| 30 | 1 | 0 | -14.60016 | 4.44377 | -4.772912 |
| 31 | 1 | 0 | -15.444578 | 6.640493 | -2.386205 |
| 32 | 1 | 0 | -8.794273 | 3.063493 | 0.203391 |
| 33 | 1 | 0 | -23.103093 | -4.292091 | 1.694453 |
| 34 | 1 | 0 | -24.637033 | -1.14819 | 1.815933 |
| 35 | 1 | 0 | -16.983076 | -6.329089 | -0.698058 |
| 36 | 1 | 0 | -20.32506 | -6.261836 | -0.691021 |
| 37 | 1 | 0 | -18.526565 | -7.762923 | 3.098556 |
| 38 | 1 | 0 | -20.347956 | -2.696908 | -3.708599 |
| 39 | 1 | 0 | -14.32678 | -0.294737 | 3.985904 |
| 40 | 1 | 0 | -17.508955 | 0.692492 | 3.831599 |
| 41 | 1 | 0 | -16.761881 | -2.543477 | 4.200641 |
| Conformer 3 | | Standard Orientation (a.u.) | | | |
| Center number | Atomic number | Atomic Type | X | Y | Z |
| 0 | 6 | 0 | -11.30729 | -1.047641 | -0.401983 |
| 1 | 6 | 0 | -11.520841 | 1.4849 | -1.732843 |
| 2 | 6 | 0 | -13.727434 | 3.128513 | -0.732262 |
| 3 | 6 | 0 | -16.165721 | 1.477718 | -0.867696 |
| 4 | 6 | 0 | -16.066615 | -1.207656 | 0.353906 |
| 5 | 6 | 0 | -13.7271 | -2.601223 | -0.670543 |
| 6 | 6 | 0 | -18.515547 | 2.83316 | -0.066455 |
| 7 | 6 | 0 | -20.659259 | 1.61057 | 0.501684 |
| 8 | 6 | 0 | -20.875814 | -1.127562 | 0.407273 |
| 9 | 6 | 0 | -18.551417 | -2.584317 | -0.500701 |
| 10 | 6 | 0 | -13.125315 | 4.210726 | 1.897013 |
| 11 | 6 | 0 | -14.074595 | 5.369249 | -2.560117 |
| 12 | 8 | 0 | -9.117836 | 2.698159 | -1.465646 |
| 13 | 6 | 0 | -23.085634 | -2.272297 | 0.936714 |
| 14 | 6 | 0 | -18.681047 | -5.417903 | 0.175515 |
| 15 | 8 | 0 | -18.872918 | -6.040959 | 2.781516 |
| 16 | 8 | 0 | -18.558949 | -2.527637 | -3.237551 |
| 17 | 1 | 0 | -16.401509 | 1.093223 | -2.902894 |
| 18 | 6 | 0 | -15.957321 | -1.127283 | 3.255426 |
| 19 | 1 | 0 | -9.714733 | -2.080183 | -1.231408 |
| 20 | 1 | 0 | -10.824917 | -0.745706 | 1.590563 |
| 21 | 1 | 0 | -11.900998 | 1.112147 | -3.755788 |
| 22 | 1 | 0 | -14.024437 | -3.052481 | -2.672931 |
| 23 | 1 | 0 | -13.484839 | -4.394376 | 0.337911 |
| 24 | 1 | 0 | -18.505724 | 4.88538 | 0.021572 |
| 25 | 1 | 0 | -22.336703 | 2.664882 | 1.05426 |
| 26 | 1 | 0 | -12.149707 | 2.864658 | 3.115819 |
| 27 | 1 | 0 | -11.880896 | 5.85182 | 1.721801 |
| 28 | 1 | 0 | -14.85507 | 4.815535 | 2.860442 |
| 29 | 1 | 0 | -15.527052 | 6.687813 | -1.906231 |
| 30 | 1 | 0 | -12.330745 | 6.479463 | -2.708547 |
| 31 | 1 | 0 | -14.602897 | 4.724389 | -4.457123 |
| 32 | 1 | 0 | -9.079902 | 4.157603 | -2.551395 |
| 33 | 1 | 0 | -23.354323 | -4.302779 | 0.831672 |
| 34 | 1 | 0 | -24.730123 | -1.156326 | 1.453825 |
| 35 | 1 | 0 | -16.972953 | -6.337732 | -0.53271 |
| 36 | 1 | 0 | -20.27068 | -6.231569 | -0.901627 |
| 37 | 1 | 0 | -20.194701 | -5.010633 | 3.504107 |
| 38 | 1 | 0 | -20.283926 | -2.78094 | -3.77178 |
| 39 | 1 | 0 | -17.577388 | -0.131706 | 4.06467 |
| 40 | 1 | 0 | -15.926421 | -3.044147 | 4.015842 |
| 41 | 1 | 0 | -14.258238 | -0.162968 | 3.901814 |
| Conformer 4 | | Standard Orientation (a.u.) | | | |
| Center number | Atomic number | Atomic Type | X | Y | Z |
| 0 | 6 | 0 | -11.152554 | -0.768059 | -0.174443 |
| 1 | 6 | 0 | -11.465578 | 1.566411 | -1.828258 |
| 2 | 6 | 0 | -13.774024 | 3.201499 | -1.087182 |
| 3 | 6 | 0 | -16.116724 | 1.419833 | -1.009832 |
| 4 | 6 | 0 | -15.906467 | -1.019374 | 0.641711 |
| 5 | 6 | 0 | -13.50455 | -2.449466 | -0.16995 |
| 6 | 6 | 0 | -18.55378 | 2.77103 | -0.514937 |
| 7 | 6 | 0 | -20.617703 | 1.59029 | 0.34977 |
| 8 | 6 | 0 | -20.664957 | -1.095933 | 0.942906 |
| 9 | 6 | 0 | -18.354344 | -2.597887 | 0.061175 |
| 10 | 6 | 0 | -13.297377 | 4.65918 | 1.379791 |
| 11 | 6 | 0 | -14.164283 | 5.161196 | -3.203947 |
| 12 | 8 | 0 | -9.174632 | 3.00337 | -1.917707 |
| 13 | 6 | 0 | -22.651174 | -2.113871 | 2.145859 |
| 14 | 6 | 0 | -18.248087 | -5.274482 | 1.191964 |
| 15 | 8 | 0 | -20.258525 | -6.740514 | 0.086025 |
| 16 | 8 | 0 | -18.485347 | -2.871841 | -2.634854 |
| 17 | 1 | 0 | -16.249541 | 0.696586 | -2.959833 |
| 18 | 6 | 0 | -15.82343 | -0.488545 | 3.499375 |
| 19 | 1 | 0 | -9.523473 | -1.844234 | -0.869107 |
| 20 | 1 | 0 | -10.678093 | -0.1844 | 1.762458 |
| 21 | 1 | 0 | -11.762353 | 0.941454 | -3.789798 |
| 22 | 1 | 0 | -13.797711 | -3.224826 | -2.071018 |
| 23 | 1 | 0 | -13.154818 | -4.047423 | 1.103495 |
| 24 | 1 | 0 | -18.661914 | 4.781086 | -0.921205 |
| 25 | 1 | 0 | -22.347788 | 2.652105 | 0.683292 |
| 26 | 1 | 0 | -12.436616 | 3.512399 | 2.866821 |
| 27 | 1 | 0 | -12.05106 | 6.273606 | 1.023268 |
| 28 | 1 | 0 | -15.07532 | 5.403049 | 2.134505 |
| 29 | 1 | 0 | -15.556237 | 6.598611 | -2.682656 |
| 30 | 1 | 0 | -12.375745 | 6.127194 | -3.581421 |
| 31 | 1 | 0 | -14.799088 | 4.255703 | -4.955625 |
| 32 | 1 | 0 | -8.709708 | 3.405142 | -0.201379 |
| 33 | 1 | 0 | -22.767567 | -4.101693 | 2.617339 |
| 34 | 1 | 0 | -24.252009 | -0.930328 | 2.654618 |
| 35 | 1 | 0 | -18.459762 | -5.227079 | 3.254213 |
| 36 | 1 | 0 | -16.42528 | -6.128609 | 0.714007 |
| 37 | 1 | 0 | -19.732514 | -8.479108 | 0.042008 |
| 38 | 1 | 0 | -19.813081 | -4.093351 | -2.934119 |
| 39 | 1 | 0 | -17.24462 | 0.896777 | 4.067862 |
| 40 | 1 | 0 | -16.161068 | -2.22147 | 4.576341 |
| 41 | 1 | 0 | -13.976717 | 0.214779 | 4.080792 |
| Conformer 5 | | Standard Orientation (a.u.) | | | |
| Center number | Atomic number | Atomic Type | X | Y | Z |
| 0 | 6 | 0 | -11.297355 | -0.976997 | -0.327074 |
| 1 | 6 | 0 | -11.509315 | 1.537441 | -1.692931 |
| 2 | 6 | 0 | -13.747034 | 3.180374 | -0.764059 |
| 3 | 6 | 0 | -16.159997 | 1.502962 | -0.956911 |
| 4 | 6 | 0 | -16.067791 | -1.138594 | 0.351994 |
| 5 | 6 | 0 | -13.706567 | -2.551294 | -0.578809 |
| 6 | 6 | 0 | -18.562883 | 2.848289 | -0.303462 |
| 7 | 6 | 0 | -20.686008 | 1.621723 | 0.331175 |
| 8 | 6 | 0 | -20.831204 | -1.120941 | 0.493871 |
| 9 | 6 | 0 | -18.539569 | -2.543233 | -0.50157 |
| 10 | 6 | 0 | -13.246969 | 4.286633 | 1.870421 |
| 11 | 6 | 0 | -14.044966 | 5.406678 | -2.615772 |
| 12 | 8 | 0 | -9.115251 | 2.768042 | -1.391309 |
| 13 | 6 | 0 | -22.920513 | -2.280529 | 1.344623 |
| 14 | 6 | 0 | -18.592806 | -5.407555 | -0.009165 |
| 15 | 8 | 0 | -18.614853 | -6.066617 | 2.608025 |
| 16 | 8 | 0 | -18.616996 | -2.425171 | -3.243525 |
| 17 | 1 | 0 | -16.300156 | 1.057541 | -2.988316 |
| 18 | 6 | 0 | -16.033502 | -0.96872 | 3.25355 |
| 19 | 1 | 0 | -9.69416 | -2.015057 | -1.128827 |
| 20 | 1 | 0 | -10.818233 | -0.635332 | 1.659058 |
| 21 | 1 | 0 | -11.846161 | 1.142843 | -3.718821 |
| 22 | 1 | 0 | -13.96493 | -3.074492 | -2.570929 |
| 23 | 1 | 0 | -13.477091 | -4.311023 | 0.493637 |
| 24 | 1 | 0 | -18.598167 | 4.900237 | -0.372934 |
| 25 | 1 | 0 | -22.389744 | 2.675164 | 0.797045 |
| 26 | 1 | 0 | -12.482373 | 2.911747 | 3.199792 |
| 27 | 1 | 0 | -11.872693 | 5.824995 | 1.742465 |
| 28 | 1 | 0 | -14.993734 | 5.045742 | 2.681602 |
| 29 | 1 | 0 | -15.490973 | 6.747997 | -1.997688 |
| 30 | 1 | 0 | -12.287462 | 6.497187 | -2.743287 |
| 31 | 1 | 0 | -14.548023 | 4.750036 | -4.51545 |
| 32 | 1 | 0 | -9.029467 | 4.163771 | -2.5546 |
| 33 | 1 | 0 | -23.042798 | -4.310424 | 1.570724 |
| 34 | 1 | 0 | -24.552427 | -1.175275 | 1.921506 |
| 35 | 1 | 0 | -16.953366 | -6.222687 | -0.985505 |
| 36 | 1 | 0 | -20.293082 | -6.139118 | -0.965161 |
| 37 | 1 | 0 | -18.463623 | -7.876997 | 2.717475 |
| 38 | 1 | 0 | -20.375583 | -2.42629 | -3.723619 |
| 39 | 1 | 0 | -14.155224 | -0.468977 | 3.936413 |
| 40 | 1 | 0 | -17.362447 | 0.445937 | 3.958939 |
| 41 | 1 | 0 | -16.527596 | -2.788838 | 4.081149 |
| Conformer 6 | | Standard Orientation (a.u.) | | | |
| Center number | Atomic number | Atomic Type | X | Y | Z |
| 0 | 6 | 0 | -11.179169 | -0.397796 | -0.253802 |
| 1 | 6 | 0 | -11.739901 | 1.842291 | -1.969568 |
| 2 | 6 | 0 | -14.107862 | 3.33409 | -1.169286 |
| 3 | 6 | 0 | -16.309439 | 1.386998 | -0.961136 |
| 4 | 6 | 0 | -15.863591 | -0.990317 | 0.732527 |
| 5 | 6 | 0 | -13.38845 | -2.25955 | -0.127541 |
| 6 | 6 | 0 | -18.816777 | 2.571421 | -0.395702 |
| 7 | 6 | 0 | -20.761925 | 1.257825 | 0.548768 |
| 8 | 6 | 0 | -20.598046 | -1.4158 | 1.175482 |
| 9 | 6 | 0 | -18.202696 | -2.761042 | 0.265748 |
| 10 | 6 | 0 | -13.633074 | 4.878634 | 1.244336 |
| 11 | 6 | 0 | -14.727488 | 5.206335 | -3.312911 |
| 12 | 8 | 0 | -9.654281 | 3.570942 | -2.030918 |
| 13 | 6 | 0 | -22.482038 | -2.561192 | 2.428016 |
| 14 | 6 | 0 | -17.863113 | -5.389155 | 1.464526 |
| 15 | 8 | 0 | -19.76949 | -7.046748 | 0.444984 |
| 16 | 8 | 0 | -18.387237 | -3.119748 | -2.417732 |
| 17 | 1 | 0 | -16.469378 | 0.609067 | -2.887625 |
| 18 | 6 | 0 | -15.727406 | -0.3877 | 3.57266 |
| 19 | 1 | 0 | -9.497921 | -1.389487 | -0.966566 |
| 20 | 1 | 0 | -10.681813 | 0.300907 | 1.632623 |
| 21 | 1 | 0 | -12.106353 | 1.11398 | -3.896291 |
| 22 | 1 | 0 | -13.686274 | -3.11057 | -1.995619 |
| 23 | 1 | 0 | -12.870537 | -3.787651 | 1.173704 |
| 24 | 1 | 0 | -19.079389 | 4.564249 | -0.813867 |
| 25 | 1 | 0 | -22.551427 | 2.198685 | 0.928799 |
| 26 | 1 | 0 | -12.591305 | 3.837324 | 2.68677 |
| 27 | 1 | 0 | -12.521738 | 6.562054 | 0.797293 |
| 28 | 1 | 0 | -15.431269 | 5.486794 | 2.071008 |
| 29 | 1 | 0 | -13.040641 | 6.306342 | -3.776202 |
| 30 | 1 | 0 | -15.354812 | 4.214682 | -5.020704 |
| 31 | 1 | 0 | -16.207882 | 6.540878 | -2.764035 |
| 32 | 1 | 0 | -8.1823 | 2.652075 | -2.58012 |
| 33 | 1 | 0 | -22.443406 | -4.545281 | 2.92767 |
| 34 | 1 | 0 | -24.156668 | -1.491634 | 2.951411 |
| 35 | 1 | 0 | -18.041304 | -5.298793 | 3.527947 |
| 36 | 1 | 0 | -15.986724 | -6.108999 | 0.974426 |
| 37 | 1 | 0 | -19.073206 | -8.722212 | 0.354372 |
| 38 | 1 | 0 | -19.631839 | -4.440627 | -2.643304 |
| 39 | 1 | 0 | -17.260999 | 0.858509 | 4.169909 |
| 40 | 1 | 0 | -15.847574 | -2.122885 | 4.691709 |
| 41 | 1 | 0 | -13.944341 | 0.516687 | 4.066845 |

**ECD Calculation Data of 3**

In general, conformational analyses of **3** were carried out via random searching in the Sybyl-X 2.0 using the MMFF94S force field with an energy cutoff of 5 kcal/mol [1]. The results showed six lowest energy conformers for both compounds. Subsequently, geometry optimizations and frequency analyses were implemented using DFT at the B3LYP-D3(BJ)/6-31G* level in the vacuum using Gaussian 09. All conformers used for property calculations in this work were characterized to be stable point on potential energy surface (PES) with no imaginary frequencies. The excitation energies, oscillator strengths, and rotational strengths (velocity) of the first 60 excited states were calculated using the TDDFT methodology at the PBE0/def2-TZVP level in PCM methanol using ORCA5.0.0. The ECD spectra were simulated by the overlapping Gaussian function (half the bandwidth at 1/e peak height, sigma = 0.30 for all). Gibbs free energies for conformers were determined by using thermal correction at B3LYP-D3(BJ)/6-31G* level and electronic energies evaluated at the wB97M-V/def2-TZVP level in PCM methanol using ORCA5.0.0 To get the ﬁnal spectra, the simulated spectra of the conformers were averaged according to the Boltzmann distribution theory and their relative Gibbs free energy (∆G). By comparing the experiment spectra with the calculated model molecules, the absolute configuration of **3** was determined.

Reference:

[1] St. Louis, MO. Sybyl Software, version X 2.0; Tripos Associates Inc. 2013.

**Figure S28.** Experimental and calculated ECD spectra of compound **3** in MeOH.

**Table S3.** Gibbs free energies^a^ and equilibrium populations^b^ of low-energy conformers of **3**.

| Conformers | ∆G (a.u.) | P(%)/100 | G (a.u.) |
| --- | --- | --- | --- |
| 1 | 0.00118 | 12.44 | -811.357821 |
| 2 | 0.00287 | 2.08 | -811.356131 |
| 3 | 0.00314 | 1.55 | -811.355857 |
| 4 | 0.00012 | 38.15 | -811.358879 |
| 5 | 0.00267 | 2.56 | -811.356329 |
| 6 | 0.0 | 43.21 | -811.358997 |

^a^PBE0/def2-TZVP, in a.u.
^b^From ∆G values at 298.15 K.

**Table S4.** Cartesian coordinates for the low-energy reoptimized random research conformers of 3*S*,5*S*,9*R*,10*S*-**3** at B3LYP-D3(BJ)/6-31G* level of theory.

| Conformer 1 | | Standard Orientation (a.u.) | | | |
| --- | --- | --- | --- | --- | --- |
| Center number | Atomic number | Atomic Type | X | Y | Z |
| 0 | 6 | 0 | -11.148716 | -0.479016 | -0.463534 |
| 1 | 6 | 0 | -11.700641 | 1.835924 | -2.08253 |
| 2 | 6 | 0 | -14.033756 | 3.341206 | -1.198209 |
| 3 | 6 | 0 | -16.256449 | 1.418285 | -1.021487 |
| 4 | 6 | 0 | -15.811012 | -1.010168 | 0.598885 |
| 5 | 6 | 0 | -13.390086 | -2.30454 | -0.359276 |
| 6 | 6 | 0 | -18.756366 | 2.603753 | -0.418432 |
| 7 | 6 | 0 | -20.697011 | 1.308762 | 0.565327 |
| 8 | 6 | 0 | -20.481287 | -1.343065 | 1.272819 |
| 9 | 6 | 0 | -18.196217 | -2.708397 | 0.139612 |
| 10 | 6 | 0 | -13.509585 | 4.797099 | 1.258324 |
| 11 | 6 | 0 | -14.647363 | 5.291395 | -3.27228 |
| 12 | 8 | 0 | -9.588735 | 3.531331 | -2.112386 |
| 13 | 6 | 0 | -22.154914 | -2.38637 | 2.870744 |
| 14 | 6 | 0 | -17.921262 | -5.435191 | 1.112684 |
| 15 | 8 | 0 | -19.837037 | -7.036795 | 0.0478 |
| 16 | 8 | 0 | -18.549623 | -2.878257 | -2.545242 |
| 17 | 1 | 0 | -16.443494 | 0.697726 | -2.967764 |
| 18 | 6 | 0 | -15.59754 | -0.494558 | 3.453244 |
| 19 | 1 | 0 | -9.50229 | -1.472391 | -1.251 |
| 20 | 1 | 0 | -10.596477 | 0.139172 | 1.435671 |
| 21 | 1 | 0 | -12.107948 | 1.186069 | -4.029308 |
| 22 | 1 | 0 | -13.749057 | -3.073533 | -2.252413 |
| 23 | 1 | 0 | -12.885003 | -3.895034 | 0.870959 |
| 24 | 1 | 0 | -19.018511 | 4.596215 | -0.840212 |
| 25 | 1 | 0 | -22.46938 | 2.264084 | 0.985544 |
| 26 | 1 | 0 | -15.287277 | 5.470556 | 2.079977 |
| 27 | 1 | 0 | -12.541151 | 3.668646 | 2.686179 |
| 28 | 1 | 0 | -12.31462 | 6.435854 | 0.865154 |
| 29 | 1 | 0 | -12.948529 | 6.379813 | -3.718148 |
| 30 | 1 | 0 | -15.305469 | 4.364371 | -5.004695 |
| 31 | 1 | 0 | -16.100932 | 6.63103 | -2.666027 |
| 32 | 1 | 0 | -8.136293 | 2.60729 | -2.703538 |
| 33 | 1 | 0 | -21.970645 | -4.279222 | 3.642047 |
| 34 | 1 | 0 | -23.748446 | -1.289612 | 3.561099 |
| 35 | 1 | 0 | -17.940536 | -5.529627 | 3.183539 |
| 36 | 1 | 0 | -16.141667 | -6.232183 | 0.437827 |
| 37 | 1 | 0 | -21.45959 | -6.463491 | 0.65521 |
| 38 | 1 | 0 | -19.538532 | -4.395926 | -2.816835 |
| 39 | 1 | 0 | -16.989143 | 0.88895 | 4.094938 |
| 40 | 1 | 0 | -15.895819 | -2.236468 | 4.528706 |
| 41 | 1 | 0 | -13.726451 | 0.205212 | 3.953897 |
| Conformer 2 | | Standard Orientation (a.u.) | | | |
| Center number | Atomic number | Atomic Type | X | Y | Z |
| 0 | 6 | 0 | -11.359607 | -1.006213 | -0.163704 |
| 1 | 6 | 0 | -11.53116 | 1.442074 | -1.669438 |
| 2 | 6 | 0 | -13.827272 | 3.10135 | -0.934885 |
| 3 | 6 | 0 | -16.214966 | 1.390982 | -1.09902 |
| 4 | 6 | 0 | -16.144856 | -1.159449 | 0.380678 |
| 5 | 6 | 0 | -13.753496 | -2.612632 | -0.400155 |
| 6 | 6 | 0 | -18.647395 | 2.755584 | -0.61594 |
| 7 | 6 | 0 | -20.776332 | 1.558392 | 0.056168 |
| 8 | 6 | 0 | -20.905575 | -1.169202 | 0.417121 |
| 9 | 6 | 0 | -18.585739 | -2.633816 | -0.443214 |
| 10 | 6 | 0 | -13.436439 | 4.380414 | 1.644198 |
| 11 | 6 | 0 | -14.051933 | 5.2106 | -2.928153 |
| 12 | 8 | 0 | -9.201027 | 2.813897 | -1.556057 |
| 13 | 6 | 0 | -22.992495 | -2.280358 | 1.333906 |
| 14 | 6 | 0 | -18.630382 | -5.463211 | 0.21974 |
| 15 | 8 | 0 | -18.654236 | -5.961572 | 2.873786 |
| 16 | 8 | 0 | -18.601096 | -2.684326 | -3.188034 |
| 17 | 1 | 0 | -16.276658 | 0.817914 | -3.101239 |
| 18 | 6 | 0 | -16.193208 | -0.796558 | 3.269395 |
| 19 | 1 | 0 | -9.723043 | -2.076341 | -0.850114 |
| 20 | 1 | 0 | -10.973746 | -0.566835 | 1.829299 |
| 21 | 1 | 0 | -11.747824 | 0.954788 | -3.680265 |
| 22 | 1 | 0 | -13.947765 | -3.237165 | -2.369926 |
| 23 | 1 | 0 | -13.552869 | -4.310148 | 0.77365 |
| 24 | 1 | 0 | -18.697303 | 4.795296 | -0.854022 |
| 25 | 1 | 0 | -22.499782 | 2.628851 | 0.395707 |
| 26 | 1 | 0 | -15.218878 | 5.176187 | 2.33276 |
| 27 | 1 | 0 | -12.736673 | 3.102817 | 3.107294 |
| 28 | 1 | 0 | -12.089961 | 5.942628 | 1.462317 |
| 29 | 1 | 0 | -12.226497 | 6.158887 | -3.135962 |
| 30 | 1 | 0 | -14.60016 | 4.44377 | -4.772912 |
| 31 | 1 | 0 | -15.444578 | 6.640493 | -2.386205 |
| 32 | 1 | 0 | -8.794273 | 3.063493 | 0.203391 |
| 33 | 1 | 0 | -23.103093 | -4.292091 | 1.694453 |
| 34 | 1 | 0 | -24.637033 | -1.14819 | 1.815933 |
| 35 | 1 | 0 | -16.983076 | -6.329089 | -0.698058 |
| 36 | 1 | 0 | -20.32506 | -6.261836 | -0.691021 |
| 37 | 1 | 0 | -18.526565 | -7.762923 | 3.098556 |
| 38 | 1 | 0 | -20.347956 | -2.696908 | -3.708599 |
| 39 | 1 | 0 | -14.32678 | -0.294737 | 3.985904 |
| 40 | 1 | 0 | -17.508955 | 0.692492 | 3.831599 |
| 41 | 1 | 0 | -16.761881 | -2.543477 | 4.200641 |
| Conformer 3 | | Standard Orientation (a.u.) | | | |
| Center number | Atomic number | Atomic Type | X | Y | Z |
| 0 | 6 | 0 | -11.30729 | -1.047641 | -0.401983 |
| 1 | 6 | 0 | -11.520841 | 1.4849 | -1.732843 |
| 2 | 6 | 0 | -13.727434 | 3.128513 | -0.732262 |
| 3 | 6 | 0 | -16.165721 | 1.477718 | -0.867696 |
| 4 | 6 | 0 | -16.066615 | -1.207656 | 0.353906 |
| 5 | 6 | 0 | -13.7271 | -2.601223 | -0.670543 |
| 6 | 6 | 0 | -18.515547 | 2.83316 | -0.066455 |
| 7 | 6 | 0 | -20.659259 | 1.61057 | 0.501684 |
| 8 | 6 | 0 | -20.875814 | -1.127562 | 0.407273 |
| 9 | 6 | 0 | -18.551417 | -2.584317 | -0.500701 |
| 10 | 6 | 0 | -13.125315 | 4.210726 | 1.897013 |
| 11 | 6 | 0 | -14.074595 | 5.369249 | -2.560117 |
| 12 | 8 | 0 | -9.117836 | 2.698159 | -1.465646 |
| 13 | 6 | 0 | -23.085634 | -2.272297 | 0.936714 |
| 14 | 6 | 0 | -18.681047 | -5.417903 | 0.175515 |
| 15 | 8 | 0 | -18.872918 | -6.040959 | 2.781516 |
| 16 | 8 | 0 | -18.558949 | -2.527637 | -3.237551 |
| 17 | 1 | 0 | -16.401509 | 1.093223 | -2.902894 |
| 18 | 6 | 0 | -15.957321 | -1.127283 | 3.255426 |
| 19 | 1 | 0 | -9.714733 | -2.080183 | -1.231408 |
| 20 | 1 | 0 | -10.824917 | -0.745706 | 1.590563 |
| 21 | 1 | 0 | -11.900998 | 1.112147 | -3.755788 |
| 22 | 1 | 0 | -14.024437 | -3.052481 | -2.672931 |
| 23 | 1 | 0 | -13.484839 | -4.394376 | 0.337911 |
| 24 | 1 | 0 | -18.505724 | 4.88538 | 0.021572 |
| 25 | 1 | 0 | -22.336703 | 2.664882 | 1.05426 |
| 26 | 1 | 0 | -12.149707 | 2.864658 | 3.115819 |
| 27 | 1 | 0 | -11.880896 | 5.85182 | 1.721801 |
| 28 | 1 | 0 | -14.85507 | 4.815535 | 2.860442 |
| 29 | 1 | 0 | -15.527052 | 6.687813 | -1.906231 |
| 30 | 1 | 0 | -12.330745 | 6.479463 | -2.708547 |
| 31 | 1 | 0 | -14.602897 | 4.724389 | -4.457123 |
| 32 | 1 | 0 | -9.079902 | 4.157603 | -2.551395 |
| 33 | 1 | 0 | -23.354323 | -4.302779 | 0.831672 |
| 34 | 1 | 0 | -24.730123 | -1.156326 | 1.453825 |
| 35 | 1 | 0 | -16.972953 | -6.337732 | -0.53271 |
| 36 | 1 | 0 | -20.27068 | -6.231569 | -0.901627 |
| 37 | 1 | 0 | -20.194701 | -5.010633 | 3.504107 |
| 38 | 1 | 0 | -20.283926 | -2.78094 | -3.77178 |
| 39 | 1 | 0 | -17.577388 | -0.131706 | 4.06467 |
| 40 | 1 | 0 | -15.926421 | -3.044147 | 4.015842 |
| 41 | 1 | 0 | -14.258238 | -0.162968 | 3.901814 |
| Conformer 4 | | Standard Orientation (a.u.) | | | |
| Center number | Atomic number | Atomic Type | X | Y | Z |
| 0 | 6 | 0 | -11.152554 | -0.768059 | -0.174443 |
| 1 | 6 | 0 | -11.465578 | 1.566411 | -1.828258 |
| 2 | 6 | 0 | -13.774024 | 3.201499 | -1.087182 |
| 3 | 6 | 0 | -16.116724 | 1.419833 | -1.009832 |
| 4 | 6 | 0 | -15.906467 | -1.019374 | 0.641711 |
| 5 | 6 | 0 | -13.50455 | -2.449466 | -0.16995 |
| 6 | 6 | 0 | -18.55378 | 2.77103 | -0.514937 |
| 7 | 6 | 0 | -20.617703 | 1.59029 | 0.34977 |
| 8 | 6 | 0 | -20.664957 | -1.095933 | 0.942906 |
| 9 | 6 | 0 | -18.354344 | -2.597887 | 0.061175 |
| 10 | 6 | 0 | -13.297377 | 4.65918 | 1.379791 |
| 11 | 6 | 0 | -14.164283 | 5.161196 | -3.203947 |
| 12 | 8 | 0 | -9.174632 | 3.00337 | -1.917707 |
| 13 | 6 | 0 | -22.651174 | -2.113871 | 2.145859 |
| 14 | 6 | 0 | -18.248087 | -5.274482 | 1.191964 |
| 15 | 8 | 0 | -20.258525 | -6.740514 | 0.086025 |
| 16 | 8 | 0 | -18.485347 | -2.871841 | -2.634854 |
| 17 | 1 | 0 | -16.249541 | 0.696586 | -2.959833 |
| 18 | 6 | 0 | -15.82343 | -0.488545 | 3.499375 |
| 19 | 1 | 0 | -9.523473 | -1.844234 | -0.869107 |
| 20 | 1 | 0 | -10.678093 | -0.1844 | 1.762458 |
| 21 | 1 | 0 | -11.762353 | 0.941454 | -3.789798 |
| 22 | 1 | 0 | -13.797711 | -3.224826 | -2.071018 |
| 23 | 1 | 0 | -13.154818 | -4.047423 | 1.103495 |
| 24 | 1 | 0 | -18.661914 | 4.781086 | -0.921205 |
| 25 | 1 | 0 | -22.347788 | 2.652105 | 0.683292 |
| 26 | 1 | 0 | -12.436616 | 3.512399 | 2.866821 |
| 27 | 1 | 0 | -12.05106 | 6.273606 | 1.023268 |
| 28 | 1 | 0 | -15.07532 | 5.403049 | 2.134505 |
| 29 | 1 | 0 | -15.556237 | 6.598611 | -2.682656 |
| 30 | 1 | 0 | -12.375745 | 6.127194 | -3.581421 |
| 31 | 1 | 0 | -14.799088 | 4.255703 | -4.955625 |
| 32 | 1 | 0 | -8.709708 | 3.405142 | -0.201379 |
| 33 | 1 | 0 | -22.767567 | -4.101693 | 2.617339 |
| 34 | 1 | 0 | -24.252009 | -0.930328 | 2.654618 |
| 35 | 1 | 0 | -18.459762 | -5.227079 | 3.254213 |
| 36 | 1 | 0 | -16.42528 | -6.128609 | 0.714007 |
| 37 | 1 | 0 | -19.732514 | -8.479108 | 0.042008 |
| 38 | 1 | 0 | -19.813081 | -4.093351 | -2.934119 |
| 39 | 1 | 0 | -17.24462 | 0.896777 | 4.067862 |
| 40 | 1 | 0 | -16.161068 | -2.22147 | 4.576341 |
| 41 | 1 | 0 | -13.976717 | 0.214779 | 4.080792 |
| Conformer 5 | | Standard Orientation (a.u.) | | | |
| Center number | Atomic number | Atomic Type | X | Y | Z |
| 0 | 6 | 0 | -11.297355 | -0.976997 | -0.327074 |
| 1 | 6 | 0 | -11.509315 | 1.537441 | -1.692931 |
| 2 | 6 | 0 | -13.747034 | 3.180374 | -0.764059 |
| 3 | 6 | 0 | -16.159997 | 1.502962 | -0.956911 |
| 4 | 6 | 0 | -16.067791 | -1.138594 | 0.351994 |
| 5 | 6 | 0 | -13.706567 | -2.551294 | -0.578809 |
| 6 | 6 | 0 | -18.562883 | 2.848289 | -0.303462 |
| 7 | 6 | 0 | -20.686008 | 1.621723 | 0.331175 |
| 8 | 6 | 0 | -20.831204 | -1.120941 | 0.493871 |
| 9 | 6 | 0 | -18.539569 | -2.543233 | -0.50157 |
| 10 | 6 | 0 | -13.246969 | 4.286633 | 1.870421 |
| 11 | 6 | 0 | -14.044966 | 5.406678 | -2.615772 |
| 12 | 8 | 0 | -9.115251 | 2.768042 | -1.391309 |
| 13 | 6 | 0 | -22.920513 | -2.280529 | 1.344623 |
| 14 | 6 | 0 | -18.592806 | -5.407555 | -0.009165 |
| 15 | 8 | 0 | -18.614853 | -6.066617 | 2.608025 |
| 16 | 8 | 0 | -18.616996 | -2.425171 | -3.243525 |
| 17 | 1 | 0 | -16.300156 | 1.057541 | -2.988316 |
| 18 | 6 | 0 | -16.033502 | -0.96872 | 3.25355 |
| 19 | 1 | 0 | -9.69416 | -2.015057 | -1.128827 |
| 20 | 1 | 0 | -10.818233 | -0.635332 | 1.659058 |
| 21 | 1 | 0 | -11.846161 | 1.142843 | -3.718821 |
| 22 | 1 | 0 | -13.96493 | -3.074492 | -2.570929 |
| 23 | 1 | 0 | -13.477091 | -4.311023 | 0.493637 |
| 24 | 1 | 0 | -18.598167 | 4.900237 | -0.372934 |
| 25 | 1 | 0 | -22.389744 | 2.675164 | 0.797045 |
| 26 | 1 | 0 | -12.482373 | 2.911747 | 3.199792 |
| 27 | 1 | 0 | -11.872693 | 5.824995 | 1.742465 |
| 28 | 1 | 0 | -14.993734 | 5.045742 | 2.681602 |
| 29 | 1 | 0 | -15.490973 | 6.747997 | -1.997688 |
| 30 | 1 | 0 | -12.287462 | 6.497187 | -2.743287 |
| 31 | 1 | 0 | -14.548023 | 4.750036 | -4.51545 |
| 32 | 1 | 0 | -9.029467 | 4.163771 | -2.5546 |
| 33 | 1 | 0 | -23.042798 | -4.310424 | 1.570724 |
| 34 | 1 | 0 | -24.552427 | -1.175275 | 1.921506 |
| 35 | 1 | 0 | -16.953366 | -6.222687 | -0.985505 |
| 36 | 1 | 0 | -20.293082 | -6.139118 | -0.965161 |
| 37 | 1 | 0 | -18.463623 | -7.876997 | 2.717475 |
| 38 | 1 | 0 | -20.375583 | -2.42629 | -3.723619 |
| 39 | 1 | 0 | -14.155224 | -0.468977 | 3.936413 |
| 40 | 1 | 0 | -17.362447 | 0.445937 | 3.958939 |
| 41 | 1 | 0 | -16.527596 | -2.788838 | 4.081149 |
| Conformer 6 | | Standard Orientation (a.u.) | | | |
| Center number | Atomic number | Atomic Type | X | Y | Z |
| 0 | 6 | 0 | -11.179169 | -0.397796 | -0.253802 |
| 1 | 6 | 0 | -11.739901 | 1.842291 | -1.969568 |
| 2 | 6 | 0 | -14.107862 | 3.33409 | -1.169286 |
| 3 | 6 | 0 | -16.309439 | 1.386998 | -0.961136 |
| 4 | 6 | 0 | -15.863591 | -0.990317 | 0.732527 |
| 5 | 6 | 0 | -13.38845 | -2.25955 | -0.127541 |
| 6 | 6 | 0 | -18.816777 | 2.571421 | -0.395702 |
| 7 | 6 | 0 | -20.761925 | 1.257825 | 0.548768 |
| 8 | 6 | 0 | -20.598046 | -1.4158 | 1.175482 |
| 9 | 6 | 0 | -18.202696 | -2.761042 | 0.265748 |
| 10 | 6 | 0 | -13.633074 | 4.878634 | 1.244336 |
| 11 | 6 | 0 | -14.727488 | 5.206335 | -3.312911 |
| 12 | 8 | 0 | -9.654281 | 3.570942 | -2.030918 |
| 13 | 6 | 0 | -22.482038 | -2.561192 | 2.428016 |
| 14 | 6 | 0 | -17.863113 | -5.389155 | 1.464526 |
| 15 | 8 | 0 | -19.76949 | -7.046748 | 0.444984 |
| 16 | 8 | 0 | -18.387237 | -3.119748 | -2.417732 |
| 17 | 1 | 0 | -16.469378 | 0.609067 | -2.887625 |
| 18 | 6 | 0 | -15.727406 | -0.3877 | 3.57266 |
| 19 | 1 | 0 | -9.497921 | -1.389487 | -0.966566 |
| 20 | 1 | 0 | -10.681813 | 0.300907 | 1.632623 |
| 21 | 1 | 0 | -12.106353 | 1.11398 | -3.896291 |
| 22 | 1 | 0 | -13.686274 | -3.11057 | -1.995619 |
| 23 | 1 | 0 | -12.870537 | -3.787651 | 1.173704 |
| 24 | 1 | 0 | -19.079389 | 4.564249 | -0.813867 |
| 25 | 1 | 0 | -22.551427 | 2.198685 | 0.928799 |
| 26 | 1 | 0 | -12.591305 | 3.837324 | 2.68677 |
| 27 | 1 | 0 | -12.521738 | 6.562054 | 0.797293 |
| 28 | 1 | 0 | -15.431269 | 5.486794 | 2.071008 |
| 29 | 1 | 0 | -13.040641 | 6.306342 | -3.776202 |
| 30 | 1 | 0 | -15.354812 | 4.214682 | -5.020704 |
| 31 | 1 | 0 | -16.207882 | 6.540878 | -2.764035 |
| 32 | 1 | 0 | -8.1823 | 2.652075 | -2.58012 |
| 33 | 1 | 0 | -22.443406 | -4.545281 | 2.92767 |
| 34 | 1 | 0 | -24.156668 | -1.491634 | 2.951411 |
| 35 | 1 | 0 | -18.041304 | -5.298793 | 3.527947 |
| 36 | 1 | 0 | -15.986724 | -6.108999 | 0.974426 |
| 37 | 1 | 0 | -19.073206 | -8.722212 | 0.354372 |
| 38 | 1 | 0 | -19.631839 | -4.440627 | -2.643304 |
| 39 | 1 | 0 | -17.260999 | 0.858509 | 4.169909 |
| 40 | 1 | 0 | -15.847574 | -2.122885 | 4.691709 |
| 41 | 1 | 0 | -13.944341 | 0.516687 | 4.066845 |
